# Radiation-Induced Morphea of the Breast in a Patient with Pre-Existing Mycosis Fungoides: A Diagnostic Challenge

**DOI:** 10.3390/curroncol33090509

**Published:** 2026-08-26

**Authors:** Tala Mobayed, Toufic Eid, Ossama Abbas, Hiba Moukadem, Nagi El Saghir, Zeina Ayoub

**Affiliations:** 1 Department of Radiation Oncology, American University of Beirut Medical Center, Beirut 1107 2020, Lebanon; tmobayed@mdanderson.org (T.M.); te04@aub.edu.lb (T.E.); 2 Department of Dermatology, American University of Beirut Medical Center, Beirut 1107 2020, Lebanon; oa09@aub.edu.lb; 3 Department of Internal Medicine, Division of Hematology-Oncology, American University of Beirut Medical Center, Beirut 1107 2020, Lebanon; hm127@aub.edu.lb (H.M.); ns23@aub.edu.lb (N.E.S.)

**Keywords:** breast cancer, radiotherapy, radiation-induced morphea, mycosis fungoides, localized scleroderma

## Abstract

Radiation-induced morphea (RIM) is a rare, immune-mediated late complication of breast radiotherapy that may clinically mimic radiation fibrosis, malignancy, or other inflammatory dermatoses. Diagnosis is particularly challenging in patients with pre-existing cutaneous T-cell lymphoma (CTCL), in whom new post-radiation skin lesions may raise concern for lymphoma involvement or progression. To the best of our knowledge, based on a non-systematic search of the available literature, this is the first reported case of radiation-induced morphea occurring in a patient with pre-existing mycosis fungoides (MF). A 55-year-old woman with a history of MF and right breast invasive lobular carcinoma underwent bilateral mastectomy followed by adjuvant chest wall radiotherapy. Approximately 6.5 years later, she developed a progressive erythematous, indurated plaque within the irradiated field. Given her history of MF, cutaneous lymphoma involvement and radiation-associated sarcoma were important diagnostic considerations. Punch biopsy demonstrated dermal collagen homogenization with a mixed inflammatory infiltrate, while immunohistochemistry showed preserved pan-T-cell antigen expression without an aberrant phenotype, supporting RIM rather than lymphoma. The patient was treated with topical corticosteroids followed by methotrexate, with mild-to-moderate improvement in erythema without complete resolution; subsequent reconstructive revision was followed by further improvement in erythema, breast softness, and cosmesis. This case highlights the diagnostic overlap between RIM and CTCL and underscores the importance of prompt biopsy and careful clinicopathological correlation in evaluating persistent or atypical post-radiation skin changes.

## Simple Summary

Radiotherapy for breast cancer can rarely cause radiation-induced morphea, in which the skin becomes thickened and hardened months to years after treatment. Diagnosis can be challenging, particularly in patients with a history of mycosis fungoides, because both conditions may appear similar. We describe a woman treated with radiotherapy for breast cancer who developed a thickened, hardened area of skin within the treated field more than six years later. Because of her history of mycosis fungoides, lymphoma involvement was initially suspected. A skin biopsy supported radiation-induced morphea rather than lymphoma. Treatment with topical corticosteroids followed by methotrexate resulted in mild-to-moderate improvement in erythema without complete resolution; subsequent reconstructive revision was followed by further improvement in erythema, breast softness, and cosmesis. This case highlights the importance of biopsy and careful evaluation of new skin changes after radiotherapy, especially in patients with a history of cutaneous lymphoma.

## 1. Introduction

Adjuvant radiotherapy following breast-conserving surgery and, in appropriately selected patients, after mastectomy reduces recurrence and breast cancer mortality [1,2]. As survivorship improves, late radiation-associated toxicities are increasingly recognized. Radiation-induced morphea (RIM) is an uncommon, delayed complication of breast irradiation, with an estimated incidence of approximately 1 in 500 to 1 in 3000 patients [3,4]. Morphea (localized scleroderma) is a localized sclerosing disorder of the dermis and subcutaneous tissue, characterized by excessive collagen deposition and fibroblast dysregulation, and is distinct from systemic sclerosis [5]. Unlike radiation-induced fibrosis (RIF), which is a late consequence of direct radiation injury, RIM is considered an immune-mediated fibroinflammatory process and may, in some patients, extend beyond the irradiated field [6,7,8]. Clinically, RIM may resemble RIF, chronic radiodermatitis, local tumor recurrence, inflammatory breast cancer, or radiation-induced sarcoma, making histopathological confirmation essential for accurate diagnosis, particularly in patients with complex oncologic or dermatologic histories in whom clinical assessment alone may be misleading [4,6].

In patients with pre-existing cutaneous disease, particularly mycosis fungoides (MF), the differential diagnosis of new post-radiation skin changes is substantially broadened. New lesions in previously irradiated fields may raise concern for lymphoma involvement or progression, creating a diagnostically complex clinical scenario. To the best of our knowledge, based on a non-systematic search of the available literature, this is the first reported case of radiation-induced morphea occurring in a patient with pre-existing mycosis fungoides. We therefore present a case in which the coexistence of MF and radiation-induced morphea created substantial diagnostic uncertainty and required histopathological evaluation to establish the diagnosis.

## 2. Case Description

### 2.1. Breast Cancer and Treatment History

The patient was 55 years old at the time of presentation with a post-radiotherapy skin lesion in 2022. She had a history of stage I mycosis fungoides (MF), a form of cutaneous T-cell lymphoma (CTCL), diagnosed in 2010 with involvement of the face and trunk. Her MF had been managed with low-dose oral methotrexate and topical therapy and subsequently came under clinical control. Methotrexate was discontinued following disease remission, and there was no clinical evidence of active MF at the time of the 2022 chest-wall presentation.

In 2015, she was diagnosed with right breast grade 1 invasive lobular carcinoma (pT1N1M0, estrogen receptor [ER]-positive, progesterone receptor [PR]-positive, human epidermal growth factor receptor 2 [HER2]-negative), associated with high-grade ductal carcinoma in situ (DCIS; 1.3 cm) and lymphovascular invasion. She underwent bilateral nipple-sparing mastectomies, including right sentinel lymph node biopsy and prophylactic contralateral mastectomy, with immediate bilateral implant-based reconstruction. Her postoperative course was complicated by a right implant infection, which required explantation prior to radiotherapy.

She subsequently received adjuvant radiotherapy (Artiste Linear Accelerator; Siemens Healthcare, Erlangen, Germany) using 6 MV photons to the right chest wall and regional lymphatics, including the supraclavicular region and axillary apex (Panther treatment planning system, version 5.10; Prowess, Concord, CA, USA). The chest wall was treated with parallel-opposed tangential fields using a single isocenter arrangement, matched to a left anterior oblique field covering the axillary apex and supraclavicular region, to 50 Gy in 25 fractions. A 5 mm bolus was applied for 15 of the 25 fractions: daily during the first 10 fractions, every other treatment during the subsequent 10 fractions, and omitted during the final 5 fractions. The skin Dmax was 52.98 Gy and D2% was 48 Gy; the global Dmax was 54 Gy (107%). A 10 Gy boost in 5 fractions was delivered to the chest-wall scar using an appositional electron field. Radiotherapy was completed in January 2016.

Approximately seven months after radiotherapy, further reconstruction was undertaken. The subsequent reconstructive course was complex and included recurrent infection and wound dehiscence; over the following years, reconstruction included a right latissimus dorsi flap, Becker expandable implant reconstruction, and additional revision procedures.

### 2.2. Post-Radiotherapy Cutaneous Presentation and Diagnostic Dilemma

In August 2022, approximately six and a half years after completing radiotherapy, the patient presented with a several-month history of intermittent erythema involving the medial aspect of the irradiated right chest wall. The erythema waxed and waned and was not associated with pain, burning, tenderness, or pruritus. There was no evidence of active mycosis fungoides at that time.

**Figure 1:**
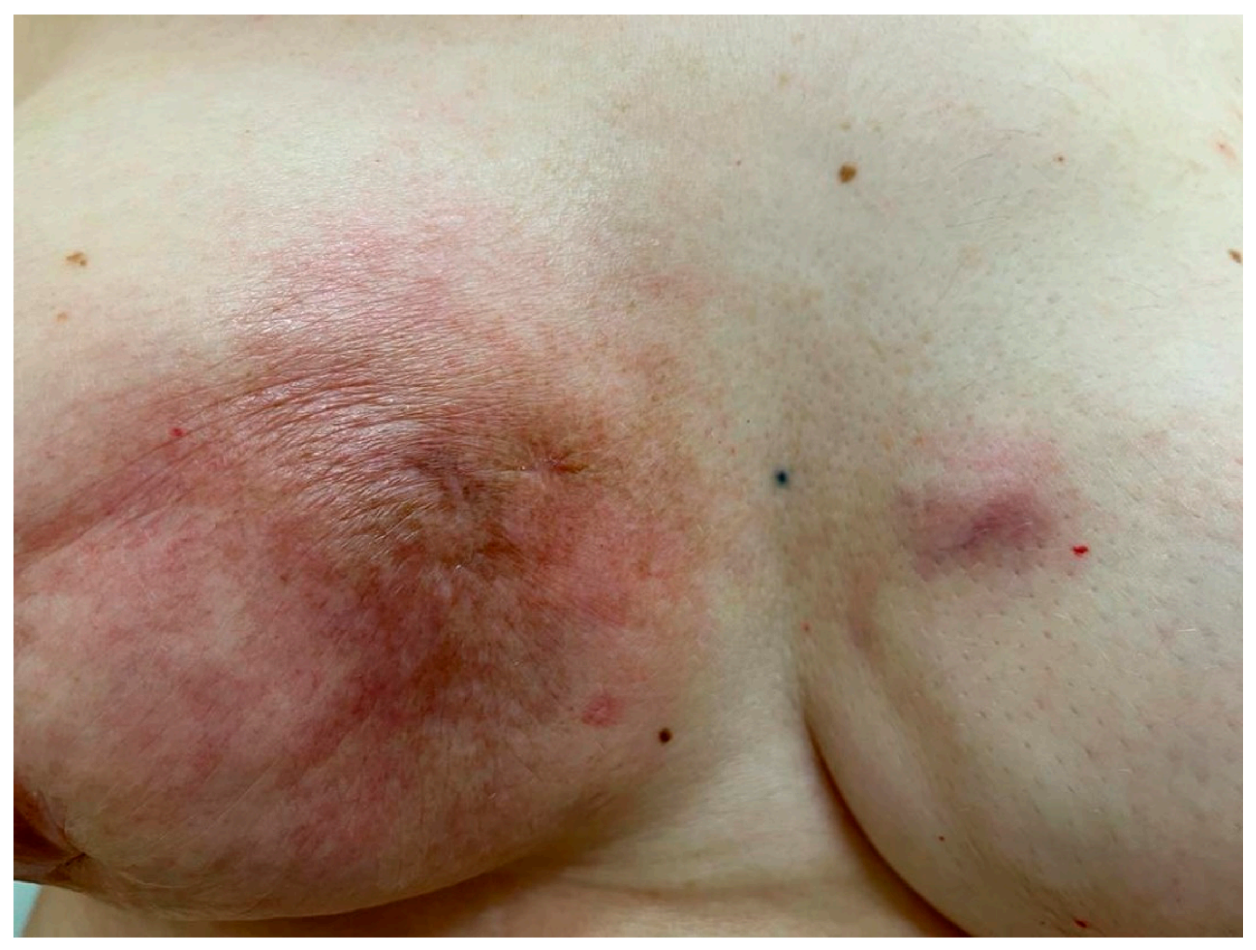
Clinical photograph of the right chest wall demonstrating an erythematous, indurated plaque within the irradiated field, with mild skin thickening, approximately six and a half years after completion of adjuvant radiotherapy.

On examination, an approximately 8 × 9 cm erythematous, indurated plaque was present within the irradiated field, with mild skin thickening over the reconstructed chest wall. The plaque was centered on native, previously irradiated skin over the medial aspect of the reconstructed right breast, medial to the preserved nipple–areolar complex, and did not involve the latissimus dorsi skin paddle. The lesion remained confined to the prior radiation field. An incidental contralateral left breast lesion, outside the radiation field, was confirmed on ultrasound to be an epidermal inclusion cyst and was unrelated to the presentation (Figure 1).

Review of the original treatment plan showed that the morphea plaque was within the irradiated and bolused chest-wall skin, in an area receiving up to approximately 52 Gy, and did not correspond to a field junction. The scar boost was located lateral to the preserved nipple–areolar complex, whereas the morphea developed medially and therefore did not overlap the boost region.

Because of her history of MF, cutaneous lymphoma involvement was considered a primary clinical concern, with chronic radiodermatitis, radiation-induced sarcoma, and radiation-induced morphea also included in the differential diagnosis. Given the patient’s history of MF and the broad differential diagnosis, tissue diagnosis was considered essential rather than relying solely on clinical appearance. Histopathological assessment with immunohistochemistry was required to distinguish inflammatory from malignant processes and to guide subsequent management, given the markedly different therapeutic implications of recurrent CTCL, radiation-associated malignancy, and RIM.

### 2.3. Histopathology

**Figure 2:**
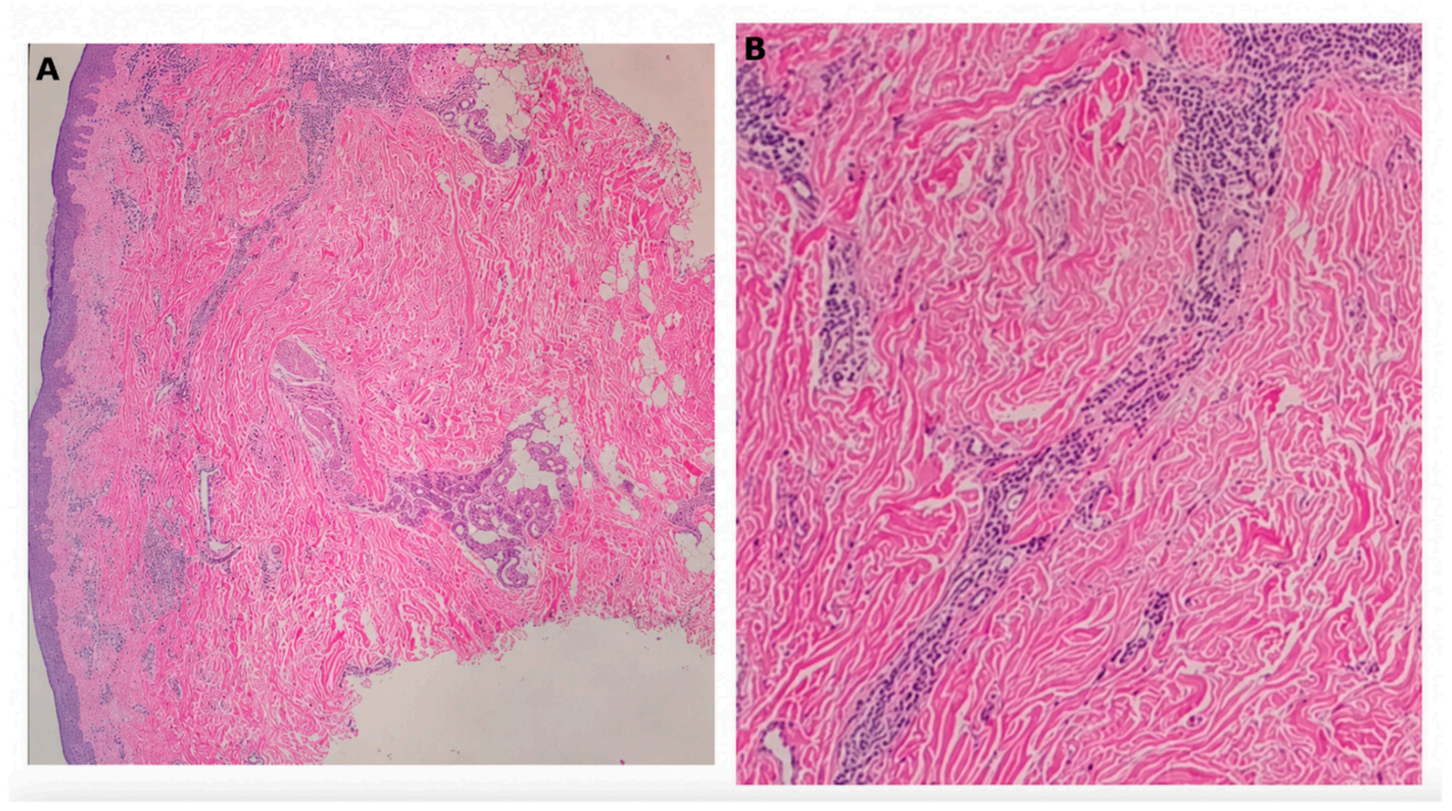
Histopathological findings of radiation-induced morphea: (**A**) Hematoxylin and eosin (H&E) staining, ×40, demonstrating diffuse dermal thickening and homogenization of collagen bundles with superficial and deep perivascular and perieccrine lymphocytic inflammation. (**B**) H&E staining, ×200, showing collagen homogenization and the inflammatory infiltrate with scattered plasma cells in greater detail.

A 5 × 5 mm punch biopsy of the right chest-wall lesion revealed diffuse dermal thickening and homogenization of collagen bundles, with a mild superficial and deep perivascular and perieccrine lymphocytic infiltrate and scattered plasma cells (Figure 2). Periodic acid–Schiff (PAS) staining was unremarkable. These findings supported a diagnosis of morphea consistent with RIM. The biopsy did not show histological features diagnostic of MF, such as epidermotropism, atypical lymphocytes, or Pautrier microabscesses. No suspicious vascular proliferation was identified on routine histology; therefore, vascular immunohistochemical studies were not considered necessary and were not performed. The sampled tissue showed no histological evidence of angiosarcoma or another radiation-associated malignancy.

Immunohistochemistry showed lymphocytes immunoreactive for the pan-T-cell markers CD2, CD3, CD5, and CD7, with a predominance of CD4-positive over CD8-positive lymphocytes. CD20 highlighted a few scattered B cells, while CD30 was negative. No aberrant loss of pan-T-cell marker expression was identified. T-cell receptor clonality testing was not performed. The 2022 biopsy was compared with the patient’s previous diagnostic MF biopsy. In conjunction with the absence of epidermotropism, atypical lymphocytes, and Pautrier microabscesses, the diffuse dermal thickening, collagen homogenization, and perieccrine lymphocytic infiltrate with scattered plasma cells favored morphea over MF involvement. Correlation of the clinical presentation with the histopathological and immunophenotypic findings was therefore essential in establishing the diagnosis. The case was discussed at the dermatology clinical meeting in August 2022, and management as radiation-induced morphea, guided by the principles applied to idiopathic localized morphea, was recommended.

### 2.4. Management and Follow-Up

The patient was treated with clobetasol propionate 0.05% cream once daily and received oral methotrexate 15 mg weekly with folic acid 5 mg weekly for approximately three months. Methotrexate had previously been used for MF and was restarted specifically for RIM. Treatment was well tolerated, with mild-to-moderate improvement in erythema without complete resolution. As the lesion was not associated with pain, pruritus, or other discomfort and she had noted improvement, the patient elected to discontinue methotrexate after three months. Reconstruction had initially been deferred because of the active skin changes. In February 2024, she underwent revision reconstruction with removal of the previous prosthesis and placement of a new implant; this was followed by further improvement in erythema, breast softness, and cosmesis.

**Figure 3:**
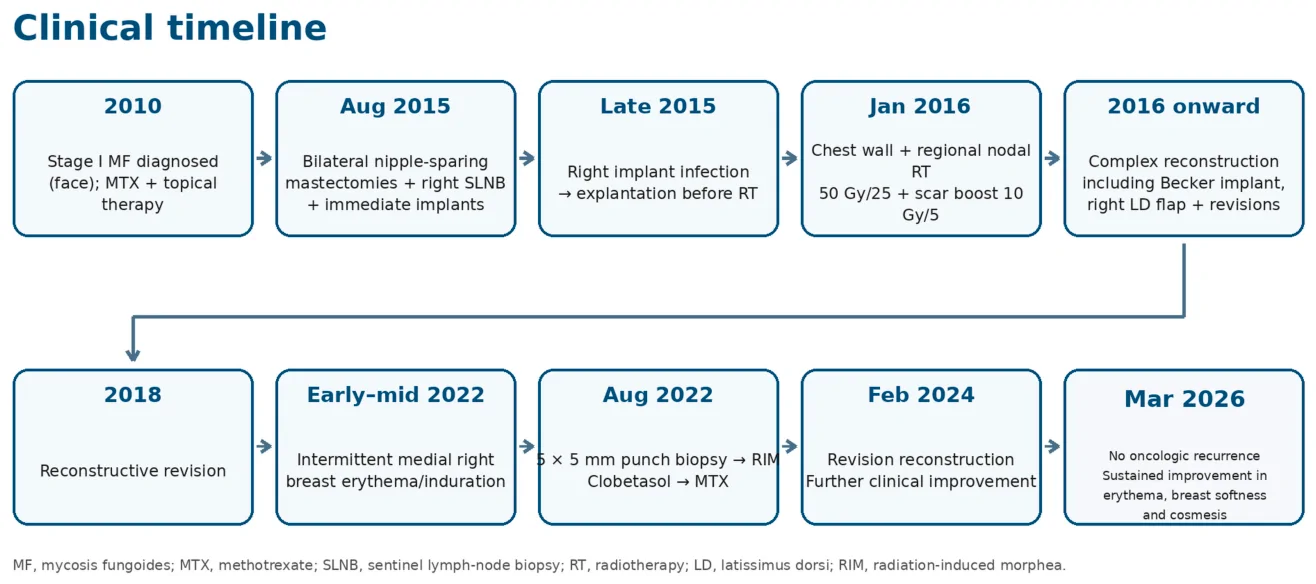
Timeline of the patient’s clinical course, from diagnosis of mycosis fungoides through breast cancer diagnosis and treatment, adjuvant radiotherapy, presentation with radiation-induced morphea, and subsequent follow-up.

At follow-up in March 2026, more than ten years after her initial breast cancer diagnosis and having completed ten years of tamoxifen therapy in October 2025, she remained free of oncologic recurrence. She remained off methotrexate, with sustained improvement in erythema, breast softness, and cosmesis (Figure 3).

## 3. Discussion

This case illustrates the diagnostic challenge of radiation-induced morphea (RIM) occurring in a patient with pre-existing mycosis fungoides (MF). The appearance of an erythematous, indurated plaque within a previously irradiated field in a patient with known cutaneous T-cell lymphoma (CTCL) raised immediate concern for lymphoma involvement or progression, as CTCL can present with erythematous, plaque-forming lesions that closely mimic both inflammatory radiation changes and morphea [9,10]. This clinical overlap rendered clinical examination alone insufficient, making histopathological evaluation essential.

Although radiation-induced morphea has been described in association with autoimmune and connective tissue disorders, its occurrence in patients with pre-existing CTCL has not been reported in major reviews and case series [3,4,6,11]. A non-systematic PubMed/MEDLINE search using combinations of the terms ‘radiation-induced morphea,’ ‘postirradiation morphea,’ ‘localized scleroderma,’ ‘mycosis fungoides,’ and ‘cutaneous T-cell lymphoma,’ together with corresponding MeSH headings, identified no previous report of radiation-induced morphea arising in a patient with pre-existing mycosis fungoides. To the best of our knowledge, this is the first reported case of this association. This association is clinically relevant given the significant clinical and histopathological overlap between morphea and MF, including reports of morphea mimicking MF [12,13]. Furthermore, CTCL lesions may clinically resemble both inflammatory radiation changes and early morphea. Therefore, new lesions arising in irradiated skin in such patients require a broad differential diagnosis, including RIM, lymphoma recurrence, chronic radiodermatitis, breast cancer recurrence, and radiation-induced sarcoma. In this case, histopathological examination supported the diagnosis of morphea and showed no histological evidence of malignancy.

Radiation-induced morphea must be distinguished from radiation-induced fibrosis (RIF), despite overlapping terminology and clinical appearance. RIF represents a late, largely non-inflammatory sequela of direct radiation-induced tissue injury, typically confined to the irradiated field and characterized by progressive induration [4,6]. In contrast, RIM is an immune-mediated fibroinflammatory process driven by endothelial injury, oxidative stress, and cytokine activation, particularly via transforming growth factor beta (TGF-β) signaling, leading to fibroblast activation and excessive collagen deposition [4,5,6,7,14]. Recent work has further characterized fibroblast dysfunction in RIM, including an aberrant myofibroblast phenotype and osteopontin as a candidate biomarker, and has reported successful treatment with repurposed mesalazine [15]. This mechanism produces an inflammatory phenotype with erythema and plaque morphology that may overlap with CTCL. Unlike RIF, RIM may extend beyond the irradiated field in some cases, supporting a distinct pathophysiology [6]. Whether immune dysregulation associated with MF predisposes to RIM remains unknown. Repeated surgery, implant infection, wound dehiscence, and reconstructive procedures represented additional local tissue insults that may have complicated the clinical interpretation of subsequent induration and fibrosis. However, the development of the plaque within native, previously irradiated skin and its confinement to the treated field supported the diagnosis of RIM.

The latency in this case (approximately 6.5 years) falls within the broad range reported in the literature. As summarized in Table 1, RIM following breast irradiation has been reported over a wide and variable latency, ranging from a few months to approximately 32 years after radiotherapy in an isolated report. The delayed presentation in our patient is therefore consistent with this broad reported latency range. 

No consistent association has been established between radiation dose, fractionation regimen, patient age, RIM occurrence, or latency [6]. Reported treatments are heterogeneous and include topical and systemic corticosteroids, methotrexate, phototherapy, and, in selected cases, surgical intervention [6,23,24].

Malignant entities must also be considered in this context. Radiation-associated sarcoma (RAS), particularly radiation-associated angiosarcoma of the breast, is a rare but serious late complication of breast radiotherapy, with a cumulative incidence of 0.1–0.3% and median latency of 6–8 years [25,26]. It typically presents as painless violaceous or bruise-like lesions that may clinically overlap with RIM and MF [26]. The similar latency periods of RAS and delayed RIM (~6–7 years) highlight the importance of biopsy and histopathological evaluation of new or evolving post-radiation skin changes [27,28]. The sampled tissue showed no histological evidence of angiosarcoma or another radiation-associated malignancy. Despite its rarity, awareness of RAS remains essential given its aggressive behavior and poor prognosis, with reported 5-year overall survival rates of approximately 35–50% [25,28].

This case also highlights therapeutic considerations. Methotrexate is an established systemic treatment for both MF and morphea [9,10,23,24] and represents a reasonable option when both conditions coexist. It is the most extensively studied systemic therapy for morphea, with evidence from randomized trials and systematic reviews [23,24]. It should be noted that the evidence supporting methotrexate in morphea derives largely from randomized and systematic-review data in idiopathic or juvenile morphea [23,24], whereas evidence specific to radiation-induced morphea is limited to case reports and small series [4,6]. Current morphea management ranges from topical treatment for limited superficial disease to methotrexate-based systemic therapy for more extensive or deeper disease [29]. Emerging targeted systemic therapies, including JAK inhibitors and tocilizumab, have been described for refractory morphea, although evidence remains limited [30]. In this patient, methotrexate had previously been used for MF and was later reintroduced for RIM, reflecting its potential utility in addressing both conditions rather than implying any effect on disease latency or clinical course.

Tamoxifen increases TGF-β activity and has been associated with radiotherapy-related pulmonary fibrosis, raising a theoretical link to fibroinflammatory processes such as RIM [4,31]. However, no significant association between anti-estrogen therapy and post-radiation morphea has been demonstrated [4], and any relationship remains speculative; its frequent co-occurrence with breast radiotherapy likely reflects confounding by indication. An open-label trial of tamoxifen for antifibrotic effect in systemic sclerosis showed no sustained benefit [32].

Clinically, this case reinforces that persistent or atypical post-radiation skin changes in patients with a history of CTCL require prompt biopsy and clinicopathological correlation. In such patients, the coexistence of MF and prior radiotherapy significantly broadens the differential diagnosis, and histological confirmation is essential to evaluate disease recurrence and malignant or inflammatory mimickers. Emerging imaging approaches, including MRI radiomics, have shown early potential for identifying patients at risk of RIM before clinical onset [22].

This report has several limitations. First, it describes a single patient and therefore cannot establish causality between radiotherapy and morphea or a causal or predisposing relationship between pre-existing MF and RIM. Second, diagnostic interpretation is limited by punch-biopsy sampling: a small punch may not capture focal atypia, deeper vascular proliferation, or heterogeneous infiltrates, limiting definitive exclusion of lymphoma involvement or angiosarcoma. Third, treatment response was documented clinically without validated morphea scoring tools, such as the modified Localized Scleroderma Skin Severity Index (mLoSSI) or Localized Scleroderma Cutaneous Assessment Tool (LoSCAT), limiting objective assessment of response. Finally, Table 1 is a non-systematic, illustrative summary rather than a systematic review; the included publications are heterogeneous in study design, radiotherapy technique, and reporting completeness, and several dose, latency, treatment, and outcome variables were not reported in the primary sources. The available evidence consists predominantly of case reports and small case series, with few cohort data; consequently, the table is intended to contextualize the present case rather than support quantitative comparisons or causal inference.

## 4. Conclusions

To the best of our knowledge, this is the first reported case of radiation-induced morphea occurring in a patient with pre-existing mycosis fungoides. The coexistence of these conditions posed significant diagnostic difficulty because the clinical presentation overlapped with both RIM and cutaneous lymphoma involvement, with additional concern for a radiation-associated malignancy. Clinical evaluation alone was insufficient to establish the diagnosis, underscoring the need for histopathological confirmation.

This case highlights the importance of prompt biopsy and meticulous clinicopathological correlation in patients with prior cutaneous lymphoma who develop atypical or persistent post-radiation skin changes. Awareness of this diagnostic overlap is essential to avoid misdiagnosis and ensure timely and appropriate management in breast cancer survivors with pre-existing CTCL.

## Figures and Tables

**Table 1:** Illustrative published reports of radiation-induced morphea following breast irradiation (non-systematic summary), with the current case.

Author/Year	Study Type	Surgery	Radiotherapy Dose/Fractionation	Latency	Extension Beyond Field	Treatment	Outcome/Notes
**Colver et al., 1989 [16]**	Case series (n = 9; 7 breast)	Breast cases: mastectomy (n = 3), local/wide excision (n = 3), NR (n = 1)	Heterogeneous; <20 to >69 Gy total; commonly 43.5–59.4 Gy in 10–27 fr; boosts in some	18 mo–10 yr	Yes (4/9)	NR	One lesion softened after 1 yr; others stable/persistent or NR. May mimic recurrence
**Bleasel et al., 1999 [17]**	Case series (n = 4)	Lumpectomy + axillary dissection (all); re-excision in 1	45 Gy/25 fr + 16 Gy/8-fr boost (3); 50.4 Gy/28 fr + 10 Gy/5-fr boost (1)	During RT or 1–3 mo after RT	No (0/4)	Topical corticosteroids under occlusion (2); intralesional steroid (1)	Two softened gradually (≈50% at 4 yr; ≈60% at 1 yr); residual induration/pigmentation
**Schaffer et al., 2000 [11]**	Two case reports + review	Simple mastectomy (n = 1); lumpectomy (n = 1)	43 Gy/20 fr, no boost (1); 46 Gy/23 fr + 18 Gy/9-fr electron boost (1)	32 yr; 6.5 yr	No (0/2)	Case 1: topical steroids + doxycycline; case 2: none	Erythema/induration improved over 9–12 mo; residual contraction in case 2
**Walsh et al., 2008 [18]**	Case series (n = 5) + review	Local excision + axillary dissection (all; all left-sided)	40–50 Gy in 15–25 fr; boosts 5–10 Gy in 3/5	4–12 yr	Yes (1/5)	Clobetasol (1); none (1); mastectomy (1); unknown (2)	One softened over 5 yr, causality uncertain; postmastectomy follow-up insufficient; others unknown
**Cheah et al., 2008 [19]**	Case report	Wide local excision + axillary sampling	40 Gy/15 fr to breast/axilla + 5 Gy/2-fr electron boost	9 mo	Probable: medial upper arm; axilla was irradiated	Antibiotics, topical/oral corticosteroids, PUVA (no benefit); analgesics; surgery declined	Progressed through 3.5 yr; stable at 6.5 yr; no cancer recurrence
**Gonzalez-Ericsson et al., 2018 [4]**	Case report + review	Segmental mastectomy with oncoplastic reconstruction + sentinel-node sampling	50 Gy/28 fr to breast/axilla; no boost	15 mo	No	Prednisone 40 mg/day + MTX 25 mg/wk + PUVA for 2 mo; MTX continued	Initial stabilization and less pain; later softer skin and resolved erythema on continued MTX
**Friedman et al., 2018 [3]**	Retrospective referral case series (n = 3; ≈12,000 treated)	Mastectomy + implant reconstruction (1); lumpectomy (2)	50 Gy/25 fr in all; boost NR	4, 5, and 7 yr	Yes in 1/3 per discussion	Steroids/topicals, MTX, UVA1, tacrolimus; 1 implant removal/skin excision for superimposed infection	No progression at 18 mo (2); complete clinical resolution at 24 mo (1). Reported prevalence ≈1:3000
**Partl et al., 2018 [20]**	Case report + review	Segmentectomy + sentinel-node dissection	50 Gy/25 fr + 10 Gy/5-fr electron boost	Onset 3 mo; diagnosis 20 mo	No (in-field)	Topical corticosteroids + lymphatic drainage; systemic corticosteroids/MTX declined	Progressive fibrosis and volume loss before diagnosis; unchanged thereafter
**Diago et al., 2019 [21]**	Retrospective case series (n = 6) + review	Breast-conserving (n = 5); mastectomy (n = 1)	46–50 Gy in 23–25 fr; boosts: 6–10 Gy (4), 7-Gy brachytherapy (1), none (1)	4–16 yr (mean 9.5 yr)	NR	High-potency topical corticosteroid (3), tacrolimus (1), UVB/MTX (1); none (2)	Treatment responses/follow-up outcomes NR; 4/6 had pre-existing autoimmune disease
**Wu et al., 2025 [22]**	Retrospective registry/proof-of-concept (n = 10; MRI subset n = 7)	Lumpectomy (n = 9); modified radical mastectomy (n = 1); immediate reconstruction (n = 1)	Mean non-boost 46.3 Gy (42.56–50.4); mean boost 10.8 Gy (10–16); boost in 9/10	Mean 24.15 mo (5.3–59.6)	NR	Medical treatments NR; total mastectomy for progression (1)	Most softened or stabilized over 3–5 yr; 7/10 biopsy-confirmed
**Current case**	Case report	Bilateral nipple-sparing mastectomies; right implant removed before RT	50 Gy/25 fr to chest wall/regional nodes + 10 Gy/5-fr electron scar boost	6.5 yr from RT to biopsy; lesion present several months before biopsy	No	Clobetasol 0.05% daily for 3 mo; MTX 15 mg/wk + folic acid 5 mg/wk for 3 mo	Mild-to-moderate improvement in erythema after medical therapy; further improvement in erythema, breast softness, and cosmesis after revision reconstruction. Pre-existing MF/CTCL

Colver et al. included two non-breast irradiation cases. Friedman et al. reported a prevalence of approximately 1:3000, although 3 cases among approximately 12,000 treatments is approximately 1:4000 [3,16]. NR = not reported; fr = fractions; MTX = methotrexate; PUVA = psoralen plus ultraviolet A; UVB = ultraviolet B; mo = months; yr = years.

## Data Availability

The data supporting the findings of this case report are included within the article. Additional information may be available from the corresponding author upon reasonable request, subject to patient confidentiality and applicable ethical considerations.

## Abbreviations

The following abbreviations are used in this manuscript:

CTCL	Cutaneous T-cell lymphoma
DCIS	Ductal carcinoma in situ
ER	Estrogen receptor
HER2	Human epidermal growth factor receptor 2
MF	Mycosis fungoides
NR	Not reported
PAS	Periodic acid–Schiff
PR	Progesterone receptor
PUVA	Psoralen plus ultraviolet A
RAS	Radiation-associated sarcoma
RIF	Radiation-induced fibrosis
RIM	Radiation-induced morphea
RT	Radiotherapy
TGF-β	Transforming growth factor beta
